# Predicting estrogen receptor status from HE-stained breast cancer slides using artificial intelligence

**DOI:** 10.3389/fmed.2025.1593143

**Published:** 2025-06-09

**Authors:** Maren Høibø, Ute Spiske, André Pedersen, Borgny Ytterhus, Lars A. Akslen, Elisabeth Wik, Cecilie Askeland, Ingerid Reinertsen, Erik Smistad, Marit Valla

**Affiliations:** ^1^Department of Clinical and Molecular Medicine, Norwegian University of Science and Technology (NTNU), Trondheim, Norway; ^2^Clinic of Laboratory Medicine, St. Olavs Hospital, Trondheim University Hospital, Trondheim, Norway; ^3^Department of Health Research, SINTEF Digital, Trondheim, Norway; ^4^Application Solutions, Sopra Steria, Trondheim, Norway; ^5^Centre for Cancer Biomarkers CCBIO, Department of Clinical Medicine, University of Bergen, Bergen, Norway; ^6^Department of Pathology, Haukeland University Hospital, Bergen, Norway; ^7^Department of Circulation and Medical Imaging, Norwegian University of Science and Technology (NTNU), Trondheim, Norway

**Keywords:** deep learning, digital pathology, breast cancer, estrogen receptor, hematoxylin and eosin, multiple instance learning, tissue microarray

## Abstract

**Introduction:**

The estrogen receptor (ER) is routinely assessed by immunohistochemistry (IHC) in breast cancer to stratify patients into therapeutic and prognostic groups. Pathology laboratories are burdened by an increased number of biopsies, and costly and resource-demanding molecular pathology analyses. Automatic, artificial intelligence-based prediction of biological properties from hematoxylin and eosin (HE)-stained slides could increase efficiency and potentially reduce costs at laboratories. The aim of this study was to develop a model for prediction of ER status from HE-stained tissue microarrays (TMAs). Our methodology can be used as proof-of-concept for the prediction of more complex and costly molecular analyses in cancer.

**Methods:**

In this study, TMAs from more than 2,000 Norwegian breast cancer patients were used to train and predict ER status using the clustering-constrained attention multiple-instance learning (CLAM) framework. Two patch sizes were evaluated, multi-branch and single-branch CLAM configurations were compared, and a comprehensive hyperparameter search with more than 16 000 experiments was performed. The models were evaluated on internal and external test sets.

**Results:**

On the internal test set, the proposed model achieved a micro accuracy, a macro accuracy, and an area under the curve of 0.91, 0.86, and 0.95, respectively. The corresponding results on the external test set were 0.93, 0.76, and 0.91, respectively. Using larger patch sizes resulted in significantly better classification performance, while no significant differences were observed when changing CLAM configurations.

## 1 Introduction

In diagnostic pathology, tissue sections are routinely stained using hematoxylin and eosin (HE) for visualization of tissue. Modern cancer diagnostics also often includes costly and complex laboratory analyses to map the biological properties of the tumor, aiming for personalized cancer treatment. The use of molecular pathology analyses has increased significantly in pathology laboratories in the past decades (1). The increased workload and recruitment difficulties pose challenges for pathology laboratories worldwide. Developing efficient and automatic methods for prediction of biological properties from hematoxylin and eosin (HE)-stained cancer slides could therefore be of clinical importance and potentially save resources in health services.

For subclassification of breast cancer into prognostic and therapeutic groups, biomarkers such as the estrogen receptor (ER) are routinely assessed according to the established breast cancer guidelines (2, 3). ER is a nuclear hormone receptor, present in benign epithelial cells and in a majority of invasive breast cancers (4), and the binding of the estrogen steroid to the ER stimulates epithelial proliferation (5). Estrogens are involved in the development and growth of breast cancer through direct and indirect mechanisms (6). ER status is determined by immunohistochemistry (IHC) (3), a method that visualizes proteins such as the ER in tissue. In the assessment of ER status, only the invasive epithelial cells are included (3). A breast cancer is classified as ER-positive if ≥ 1% of the invasive epithelial cells have ER-positive nuclei, regardless of staining intensity (3, 7). Determining ER status is important, as ER-positive patients may benefit from endocrine therapy (2), improving their prognosis (8, 9). There are limited data on the effect of endocrine therapy for the 1%–10% ER-positive (ER low) group. However, only approximately 2%–3% of breast cancer patients have tumors in this category (3). Less than 10% of normal breast epithelial cells are ER-positive (10), while approximately 70%–80% of breast cancers are ER-positive (8, 11). ER-negative breast cancers are associated with more aggressive clinical behavior than ER-positive breast cancers (12, 13). While ER-negative breast cancers are a heterogeneous group, they are associated with morphological features such as comedo-type necrosis, lymphoid stroma, pushing margins, and histological grade III (14). The proportion of ER-negative breast cancers varies between different histological subtypes. Cancers with medullary features and metaplastic carcinomas, are, for instance, often ER-negative (15, 16), whereas invasive lobular carcinomas are often ER-positive (17).

With the introduction of digital pathology, artificial intelligence (AI) has become an emerging field in diagnostic pathology (1). It has been used for tasks such as segmentation, detection, and prediction of prognosis and biological properties in cancer (18–22). However, the extreme size of histopathological images, with sizes up to 200,000 × 100,000 pixels, poses several technical AI challenges. In diagnostics, breast cancer tumors are subclassified based on biomarker status, resulting in patient-level labels. However, as pathology images are large, patch-based methods are commonly used in AI analysis of digital tissue sections.

Convolutional neural networks (CNNs) are widely used in traditional image analysis due to their ability to automatically learn spatial features from raw pixel data. While highly effective in many computer vision tasks, their direct application for class prediction in digital pathology presents challenges due to the large image size (23). In patch-based approaches, CNNs process individual image patches and aggregate predictions uniformly, which may dilute critical signals, especially if only a small subset of image regions reflect the diagnostic label. This limitation becomes especially pronounced in heterogeneous slides where applying a slide-level label to all patches can lead to suboptimal performance (22, 24, 25). It is challenging to predict patient-level labels from small image patches, as the morphology in each patch may not reflect the given label. For instance, some patches may contain invasive epithelial cells, while others contain only adipose tissue, fibrous tissue, or inflammatory cells. Multiple instance learning (MIL) is a weakly supervised technique that can be used to solve the challenge of patch-based classification on tumors with intratumor morphological heterogeneity. In MIL, bags of instances, for example image patches or patch features, are labeled and used for training instead of individual instances. The bag can, for example, be labeled as positive if one instance within the bag is positive, and negative otherwise. The model will learn to separate positive and negative instances based on the bag labels. Attention MIL further addresses these challenges by allowing the model to weigh patches differently through attention mechanisms while only requiring slide-level labels (26). Advanced MIL variants such as clustering-constrained attention multiple instance learning (CLAM) incorporate clustering-based contextual learning to further enhance the performance in complex tissue landscapes (27).

The prediction of biomarkers from scanned tissue slides is a rapidly evolving area in cancer research (28–33). Prediction of ER status from HE-stained breast cancer slides has also been attempted previously. Couture et al. used a truncated VGG16 CNN, pre-trained on ImageNet, with the addition of a custom classifier consisting of a support vector machine to predict ER status, tumor grade, basal-like vs. non-basal-like, ductal vs. lobular, and risk of recurrence score in tissue microarrays (TMAs) from breast cancer tumors (28). They trained on a set of 571 patients and evaluated on a test set of 288 patients, with accuracy, recall, and specificity of 84%, 88%, and 76%, respectively, using an ER cutoff of 10% (28). While they achieved high accuracy, specificity, and recall, it is possible the model would benefit from a CNN pretrained on HE-stained slides, and not ImageNet. Akbarnejad et al. predicted the proliferation marker Ki-67, ER status, progesterone receptor (PR) status, and human epidermal growth factor receptor 2 (HER2) status from HE images using patch-level labels from 59 whole slide images (WSIs) with a ResNet-18 and a vision transformer, and WSI labels with CLAM (31). The ground truth ER statuses were automatically generated from HE and IHC WSIs, making it difficult to compare with models trained on manually assessed ER statuses. They achieved a median area under the curve (AUC) of approximately 0.70 for ER prediction with CLAM. With a vision transformer pipeline for patch classification, they achieved a higher AUC. Wang et al. predicted ER, PR, and HER2 status in WSIs of HE-stained slides with a multi-label model. For ER status, they achieved AUCs of 0.88 and 0.92 and accuracies of 0.81 and 0.85 on two different datasets (*n* = 757 and *n* = 2,384) (34), which indicates that the dataset may influence the results. To counter this, Wang et al. did a comparison of multiple MIL models on the two datasets, where their model achieved the highest AUC score for ER status and the third highest accuracy on both datasets (34). Gamble et al. predicted ER, PR, and HER2 in HE-stained slides achieving an AUC of 0.86 (32). They found that ER-negative breast cancers were associated with tumor-infiltrating lymphocytes and ER-positive cancers with low histological grade. Tafavvoghi et al. (35) used a two-stage approach to classify HE-stained breast cancer slides into molecular subtypes. They first classified patches as tumorous or non-tumorous and then used the tumorous patches only for classification of molecular subtypes. Feature extraction in digital pathology involves transforming raw WSIs, or patches of these, into meaningful representations that capture tissue morphology and histopathological patterns. In a comprehensive benchmark study of 14 feature extractors studying their performance on nine downstream tasks, UNI (27), Lunit-DINO (36), and CTransPath (37) outperformed the 11 other feature extractors, including Swin (38), Vit-B (39), and ResNet-50 (40), on all downstream tasks (41). The three leading feature extractors also demonstrated robustness to stain variations and augmentations in contrast to ImageNet baselines.

The aim of this study was to predict ER status in HE-stained breast cancer TMAs using CLAM (27) with the vision-encoder UNI (42). A thorough hyperparameter search was performed to tailor CLAM to ER prediction in breast cancer tumors, and six classification heads and two patch sizes were evaluated to find the best configuration. The model was evaluated on two independent test sets. Such methods can be used as proof-of-concept for the prediction of other biological properties in cancer. The source code to reproduce the experiments is available at https://github.com/AICAN-Research/estrogen-receptor-prediction.

## 2 Materials and methods

### 2.1 Dataset

**Figure 1:**
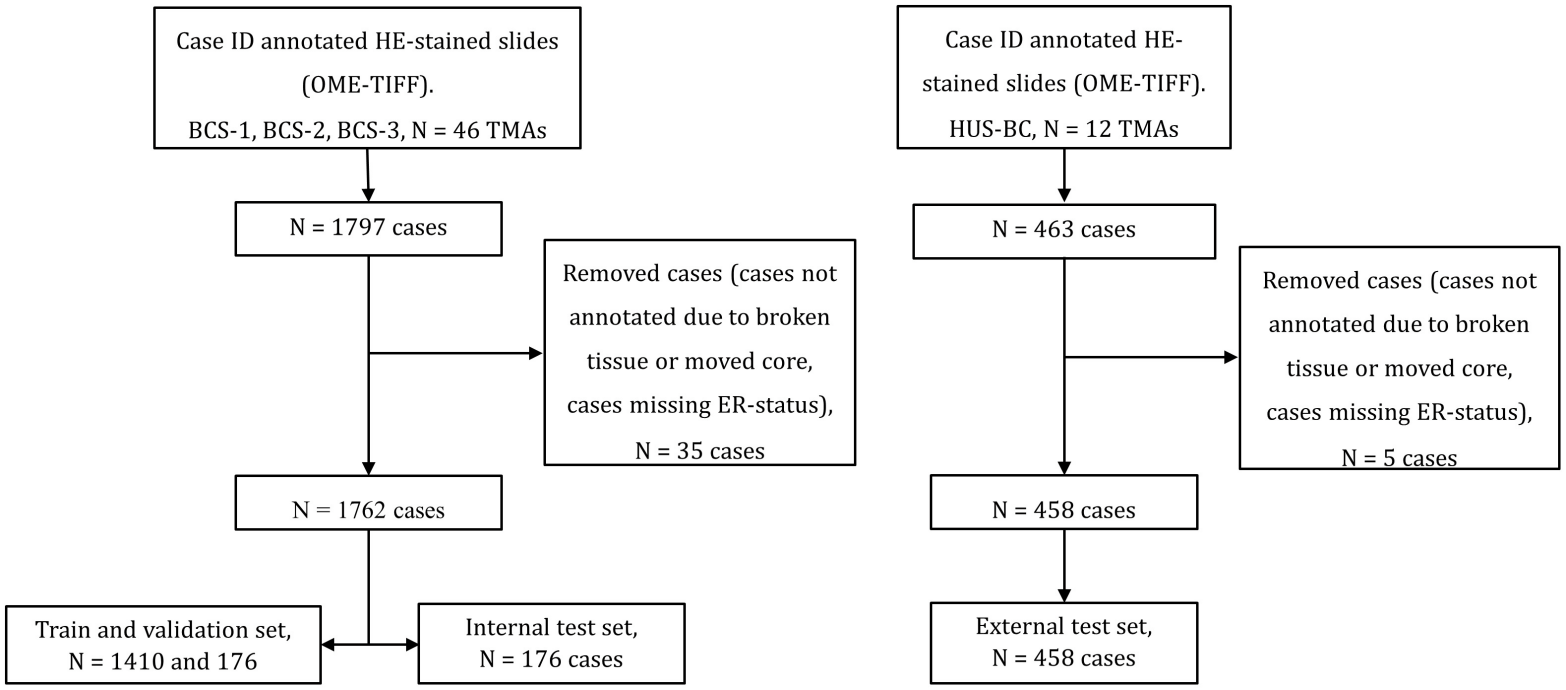
Data selection flow. **Left**: BCS-1, BCS-2, and BCS-3 were used for training, validation, and an internal test set. **Right**: HUS-BC was used as an external test set. A total of 35 and 5 patients were removed from BCS-1, BCS-2, and BCS-3 and HUS-BC, respectively, due to broken or shifted cores, or missing estrogen receptor (ER) status.

The dataset includes TMAs from four breast cancer cohorts:

- BCS-1: In 1956-1959, a population-based survey was conducted in three counties in Norway (43). BCS-1 includes women from the county for Nord-Trøndelag who were invited to participate in this survey. The cohort comprises a background population of 25,727 women who were followed for breast cancer occurrence from 1961 to 2008 (44). The women were born between 1886 and 1928. Breast cancer tumors from 909 of these women were reclassified into molecular subtypes (45), and in the present study, we included 22 TMAs, comprising a total of 890 patients from this cohort. A total of 25 patients were excluded during preprocessing due to broken tissue, moved cores, or missing ER status (Figure 1). Thus, 865 patients were included in our study. This is described in more detail in Section 2.2.
- BCS-2: Between 1995 and 1997, a health survey was conducted in Nord-Trnødelag County, Norway (46). BCS-2 includes a background population of 34,221 women, born 1897-1977, who were followed for breast cancer occurrence from attendance in the survey, until 2009. Breast cancer tumors from 514 of these women were previously reclassified into molecular subtypes (44). In the present study, we included 12 TMAs, comprising a total of 438 patients from this cohort. During preprocessing, seven patients were excluded due to broken tissue, moved TMA cores, or missing ER status. Thus, 431 patients from BCS-2 were included in our study.
- BCS-3: A total of 22 931 women born at EC Dahl's Foundation, Trondheim, Norway, between 1920 and 1966 were followed for breast cancer occurrence between 1961 and 2012 (47). Breast cancer tumors from 533 of these women were previously reclassified into molecular subtypes (48). In the present study, we included 12 TMAs comprising a total of 469 patients from this cohort. In total, three patients were excluded due to broken tissue, moved TMA cores, or missing ER status. Thus, 466 patients from BCS-3 were included in our study.
- HUS-BC includes 534 women with breast cancer, diagnosed through the National Breast Cancer Screening Program in Hordaland County, Norway, between 1996 and 2003 (49). The women were between 50 and 69 years old at diagnosis. In case of distant metastasis at the time of diagnosis, patients were not included (49). In the present study, we included 12 TMAs, comprising a total of 463 patients from this cohort. Due to broken tissue, moved TMA cores, or missing ER status, five patients were excluded during preprocessing. Thus, 458 patients from HUS-BC were included in our study.

The TMAs from BCS-1, BCS-2, and BCS-3 were made in 2011, 2014, and 2016, respectively, whereas the TMAs from HUS-BC were made in 2004 (50) and 2012 (49). All slides were HE-stained and scanned at × 40 at the Norwegian University of Science and Technology (NTNU) using Olympus VS120 and Olympus VS200 scanners. Extended focal imaging was used while scanning 12 of the slides from BCS-1, BCS-2, and BCS-3 and when scanning all slides from HUS-BC.

The TMAs from BCS-1, BCS-2, and BCS-3 comprised 1-3 cores from each tumor. Each core had a diameter of 1 mm, and the TMA slides were 4 μm thick. The TMAs from HUS-BC comprised 1-6 cores from each tumor. Each core had a diameter of 0.6 (50) or 1 mm (49), and the TMA slides were 5 μm thick. ER status was assessed using IHC in 2012, 2014, and 2016-2017 for BCS-1, BCS-2, and BCS-3, respectively. A cutoff of 1% was used (44, 45, 48). The choice of ER cutoff was based on international guidelines (3, 7) ER status for HUS-BS was originally collected from clinical records, using a cutoff of 10% (49). A new assessment of ER status using a 1% cutoff was later performed in 2019 (previously unpublished data).

### 2.2 Preprocessing

**Figure 2:**
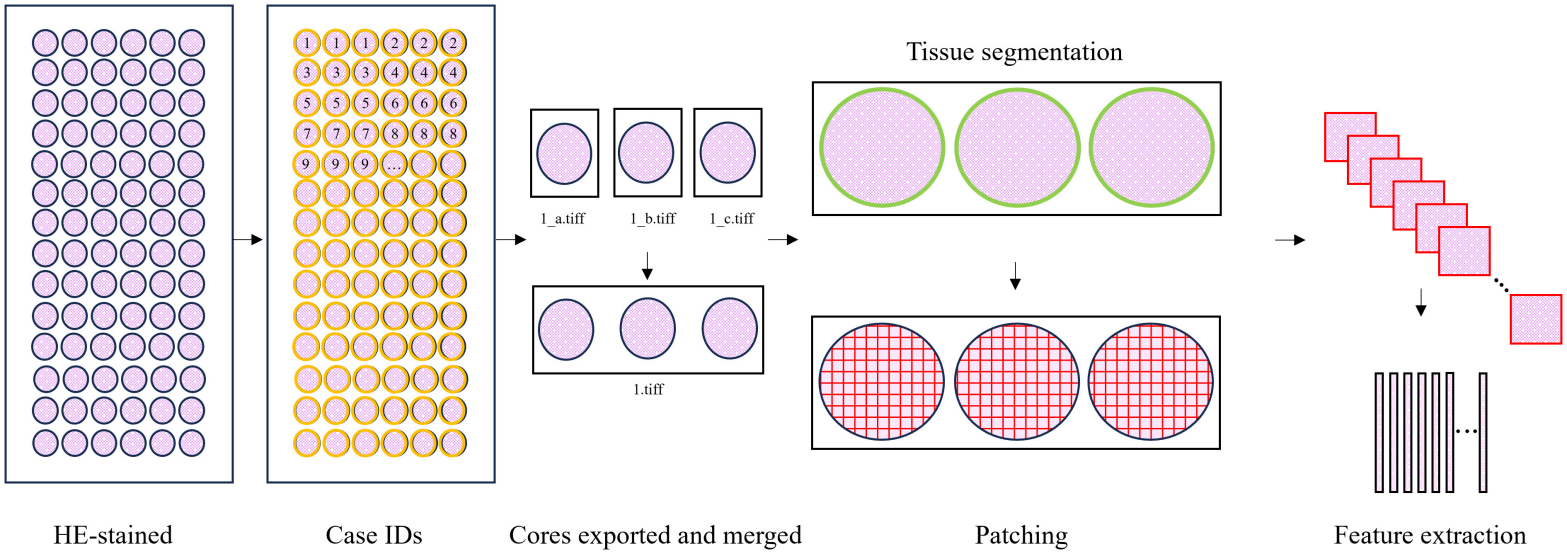
Preprocessing. The tissue microarray (TMA) slides were HE-stained. The patient IDs were annotated in QuPath. Each TMA core was exported as a single TIFF image using QuPath. The TMA cores from the same patient were merged into one TIFF image. The images were then patched, and their features were extracted using CLAM (27).

First, the TMAs were converted to the OME-TIFF format using the bioformats2raw and raw2ometiff tools by Glencoe Software (51). The TMA cores were then annotated in QuPath (52), linking each core to its respective patient ID (Figure 2). The TMA cores were then exported as separate TIFF images at magnification × 40 using QuPath. Since a tumor's ER status is determined based on ER expression in all of its TMA cores, the TMA core images from each patient were merged into one image per patient (Figure 2). This resulted in 1770 images from 1770 patients from BCS-1, BCS-2, and BCS-3. ER status for eight patients was missing, thus 1762 patients with ER status were included in the study. Of the 1762 included patients, 1473 were ER-positive and 289 were ER-negative (Supplementary Table S1). The TMA core extraction, and combination of cores into one image per patient, resulted in 458 images with ER status for HUS-BC (Figure 1).

The data were then split on patient level. The BCS-1, BCS-2, and BCS-3 cohorts were merged to form the internal dataset. A subset of 10% of the TMAs was randomly extracted from the three cohorts to form the internal test set. The fourth cohort, HUS-BC, was used as an external test set. The proportions of ER-negative samples in the internal and external test sets were 16% and 13%, respectively (Table 1).

**Table 1:** Data description.

	**Cohorts**				**ER status**		
	**BCS-1**	**BCS-2**	**BCS-3**	**HUS-BC**	**ER**+	**ER-**	**Total**
**Histological subtype**							
Ductal	606	339	401	383	1484	245	1,729
Lobular	118	48	27	48	223	18	241
Medullary	22	17	20	4	20	43	63
Mucinous	36	14	8	14	69	3	72
Papillary	31	5	0	0	32	4	36
Metaplastic	12	3	1	0	2	14	16
Tubular	3	0	2	6	11	0	11
Other	36	5	7	3	30	21	51
Missing	1	0	0	0	1	0	1
**Histological grade**							
I	102	84	55	186	416	11	427
II	461	214	227	196	1,017	81	1,098
III	300	133	184	76	437	256	693
Missing	2	0	0	0	2	0	2
**Total**	865	431	466	458	1872	348	2,220

Histological subtype and histological grade of patients included in BCS-1, BCS-2, BCS-3, and HUS-BC. Estrogen receptor (ER) status for all four cohorts combined.

Tissue segmentation was then performed using CLAM with a custom preset file (Supplementary Table S5). Each TMA core was then divided into image patches from within the tissue area with CLAM, and features were extracted from the individual image patches and stored on disk using UNI (Figure 2).

### 2.3 Classification approach

CLAM (27) is a framework for training WSI-level classifiers using slide-level labels. It uses features extracted with an encoder as input. CLAM is based on MIL, where a MIL bag consists of features of patches from one image, and an instance is the features from a single image patch. CLAM uses attention to identify important features within a bag for the given classes. During training, a clustering step is used to separate the most, and least, important features (high and low attention) for each class in a bag. CLAM can be used with single-branch attention or multi-branch attention. Single-branch attention is used for binary classification tasks, whereas multi-branch attention is best for multi-class classification or complex binary classification tasks, where each class is assigned an attention branch.

UNI (42) is a general-purpose self-supervised foundation model for pathology that can be used to extract biologically meaningful features from histopathology images. It generates a unified feature space that captures tissue morphology across diverse cancer types.

In this study, CLAM was used to predict ER status from TMA cores. Image patch features were extracted with size 1,024 × 1,024 from magnification × 40 using the UNI (42) encoder. Features from TMA cores from the same patient ID were stored together in a bag. Each bag was then assigned using the corresponding TMA-level label. These labeled bags were then used to train CLAM. Multi-branch class-wise attention was enabled, with one attention branch for each class. To counter class imbalance, CLAM's weighted sampling scheme was used during training. The final output was a slide-level attention vector, which was combined with the nano classification head (number of neurons in each hidden layer: {1,024, 512, 128, 64, 32}) to generate the final predictions (Supplementary Tables S3, S4).

### 2.4 Experiments

To assess the impact of individual components in CLAM and determine the best performing design, an exhaustive hyperparameter search was performed. Monte Carlo cross-validation with *k* = 10 splits was used for model training. This split was kept fixed for all experiments.

**Figure 3:**
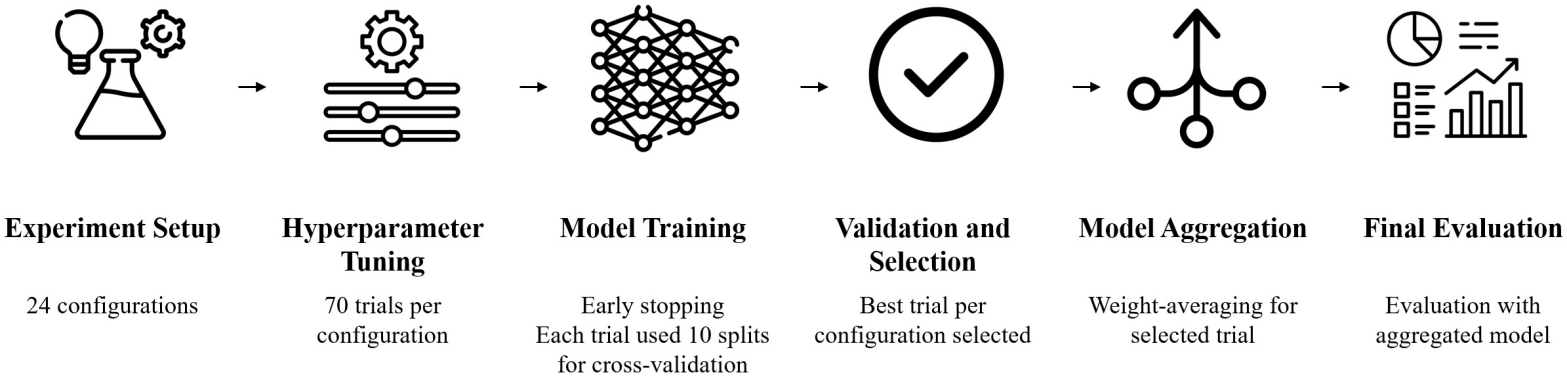
Experiment workflow. A total of 70 trials for 24 different configurations of CLAM were performed. The best trial per configuration was selected, and the final best models per configuration were selected through averaging weights from respective cross-validation runs. The final 24 aggregated models were then evaluated on two separate test sets.

A total of 24 different configurations of CLAM were tested (Figure 3). The following components were evaluated: patch size, classification head, and CLAM design (multi-branch and single-branch). Patch sizes of 256 × 256 and 1,024 × 1,024 were compared to investigate whether patch size affects performance. Six different classifier heads were compared. Finally, the impact of using a single attention branch (single-branch) or one attention branch per class was tested (multi-branch) due to the high complexity of the binary classification task.

For each configuration, 70 hyperparameter trials were performed. Overall, a total of 24 × 70 × 10 = 16,800 separate models were trained. Each model was trained using early stopping with a patience of 20 epochs. Only trials where at least one split ran for more than 70 epochs were kept to ensure that the model converged and learned meaningful patterns. The hyperparameter search was performed using the Optuna (53) framework. To give the minority class a high priority during the hyperparameter search, it was optimized toward a weighted class accuracy. The class weights were determined from the inverse class frequency, resulting in weights of 0.8 and 0.2 for the ER-positive and ER-negative classes, respectively.

Due to class imbalance, the weighted class accuracy (Supplementary Equation S1), the sum of the ER-positive class accuracy multiplied by 0.2 and the ER-negative class accuracy multiplied by 0.8, was then calculated for each of the configurations and averaged across the 10 folds. For each of the 24 configurations, the trial that resulted in the highest average weighted accuracy was kept. The final model was created using weight averaging, where the weights of the 10 model splits were aggregated and averaged to form a single, more robust model. The final 24 models were then evaluated on the internal and external test sets.

The micro accuracy, macro accuracy (Supplementary Equation S2), and AUC for the two test sets were calculated using the averaged model for each of the 24 configurations. The micro accuracy is the sum of the true-positive cases (correctly predicted ER-positive patients) and true-negative cases (correctly predicted ER-negative patients) divided by the number of patients. The macro accuracy is the average of the two class accuracies. A tumor was classified as ER positive when the model predicted a positive tumor with a probability of 0.50 or greater. The best performing model, based on weighted accuracy, on the internal test set was used to generate heatmaps for the internal test set and calculate the true positives, false positives, true negatives, and false negatives for the two test sets by histological subtype and histological grade.

The data were further analyzed in the feature space by visualizing the two main components of the slide-level features obtained from a two-component principal component analysis (PCA). The PCA transformation was computed using the training and validation feature sets, capturing their variation. This transform was then applied to both test sets.

Statistical analysis was conducted to determine whether any of the following factors impacted classification accuracy: patch size, classification head, CLAM design (single-branch or multi-branch), and test set. A binomial logistic regression model was used, treating the classification accuracy as a dependent variable and the four remaining variables as independent factor variables. Independent variables were checked for multicollinearity, and all had a variance inflation factor of less than 2, indicating negligible multicollinearity.

TMA cores were extracted using QuPath v0.5.1 (52). All experiments were conducted in Python 3.10.12. Model training was performed using PyTorch v2.3.0 (54), hyperparameter tuning using Optuna v3.6.1 (53), and statistical analysis using statsmodels v0.14.4 (55). The experiments were carried out on an Intel Xeon Gold 6239 central processing unit (CPU), using a dedicated Quadro RTX 6000 NVIDIA graphics processing unit (GPU), 256 GB RAM, and a regular hard drive.

This study was approved by the Regional Committee for Medical Research Ethics Central Norway (2018/2141). The need for consent was waived.

## 3 Results

The best performing model was a CLAM multi-branch model trained on patch size 1,024 × 1,024 with a nano classification head. Patch size 1,024 × 1,024 was significantly better than patch size 256 × 256, while there was no overall significant difference between CLAM multi-branch and CLAM single-branch models, nor between the different classification heads. The best performing models tended to have configurations with low dropout rates and favored SGD as the optimizer (Supplementary Table S9).

The proposed method achieved macro accuracies of 0.86 and 0.76 on the internal and external test sets and weighted accuracies of 0.82 and 0.52 on the internal and external test sets, respectively (Table 2 and Supplementary Table S6). The ER-negative class accuracy and the ER-positive class accuracy on the internal test set were 0.79 and 0.93, respectively, whereas on the external test set, the ER-negative class accuracy was 0.53 and the ER-positive class accuracy was 0.99 (Supplementary Table S6). The micro accuracy and AUC on the internal and external test sets were 0.91 and 0.95, and 0.93 and 0.91, respectively.

**Table 2:** Performance metrics.

	**Configurations**			**Internal test set**			**External test set**		
	**Method**	**Patch size**	**Classifier**	**ACC**	**mACC**	**AUC**	**ACC**	**mACC**	**AUC**
(1)	sb	256	Big	0.881	0.749	0.930	0.902	0.648	0.897
(2)	sb	256	Small	0.898	0.745	0.924	0.906	0.657	0.890
(3)	sb	256	Mini	0.892	0.783	0.930	0.906	0.664	0.898
(4)	sb	256	Micro	0.898	0.773	0.928	0.908	0.666	0.897
(5)	sb	256	Nano	0.875	0.773	0.914	0.902	0.655	0.887
(6)	sb	256	Pico	0.881	0.804	0.919	0.906	0.664	0.893
(7)	mb	256	Big	0.881	0.790	0.911	0.908	0.659	0.886
(8)	mb	256	Small	0.892	0.783	0.921	0.913	0.675	0.895
(9)	mb	256	Mini	0.869	0.770	0.917	0.910	0.667	0.891
(10)	mb	256	Micro	0.875	0.773	0.917	0.908	0.659	0.890
(11)	mb	256	Nano	0.875	0.773	0.916	0.908	0.666	0.887
(12)	mb	256	Pico	0.881	0.762	0.921	0.910	0.674	0.890
(13)	sb	1,024	Big	0.909	0.835	**0.952**	0.926	0.741	**0.922**
(14)	sb	1,024	Small	**0.915**	0.852	0.951	0.928	0.764	0.916
(15)	sb	1,024	Mini	0.909	0.849	0.947	0.924	0.739	0.912
(16)	sb	1,024	Micro	**0.915**	0.852	0.951	0.928	0.764	0.917
(17)	sb	1,024	Nano	0.909	0.849	**0.952**	0.926	0.748	0.916
(18)	sb	1,024	Pico	**0.915**	0.852	0.950	0.924	0.739	0.907
(19)	mb	1,024	Big	0.898	0.814	0.947	0.924	0.732	0.914
(20)	mb	1,024	Small	0.909	0.835	0.946	0.924	0.747	0.915
(21)	mb	1,024	Mini	0.909	0.835	0.947	0.924	0.739	0.916
(22)	mb	1,024	Micro	**0.915**	0.852	0.948	**0.932**	**0.773**	0.918
(23)	mb	1,024	Nano	0.909	**0.863**	0.951	0.928	0.756	0.915
(24)	mb	1,024	Pico	0.898	0.787	0.945	0.906	0.664	0.902

Results for each configuration on the internal test set and the external test set. SB, single-branch; MB, multi-branch; ACC, accuracy; mACC, macro accuracy; AUC, area under the curve. The bold values represent the highest value for each column.

**Figure 4:**
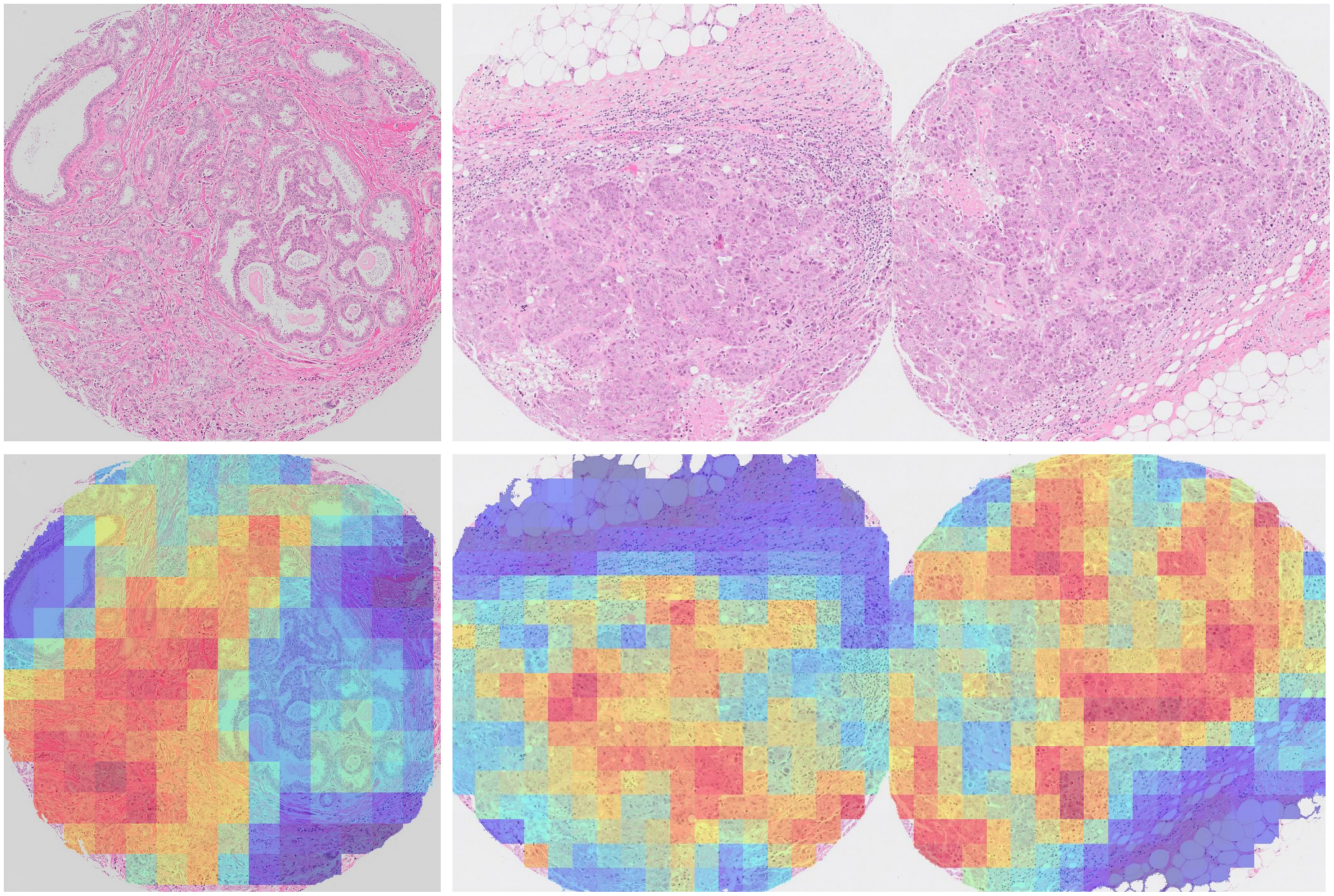
Attention heatmaps of TMA cores from two breast cancer tumors from the internal test set where ER status was correctly predicted. **Left**: ductal carcinoma, histological grade I, ER-positive tumor. The high attention (red) areas are mainly found among invasive epithelial cells. Non-invasive epithelial cells are found in low attention areas (blue). **Right**: ductal carcinoma, histological grade III, ER-negative tumor. The high attention (red) areas are mainly found among invasive epithelial cells, while connective and adipose tissues are found in low attention areas (blue).

The five correctly predicted ER-negative tumors with the highest probability scores in the internal test set had histological grade III and medullary or ductal histological subtypes. ER status was correctly predicted with high confidence and the model focused on invasive epithelial cells in the generated heatmaps from the internal test set in Figure 4. Five of the seven medullary tumors in the internal test set were correctly predicted as ER-negative tumors, while one was correctly predicted as ER-positive, and one incorrectly predicted as ER-negative. In the external test set, three of the four medullary tumors were correctly predicted as ER-negative tumors, whereas one was incorrectly predicted as ER-positive tumors (Supplementary Table S7).

In the internal test set, 93.2% and 79.3% of the ER-positive and ER-negative tumors were correctly predicted as ER-positive and ER-negative tumors, respectively. In the external test set, 98.7% and 52.5% of the ER-positive and ER-negative tumors were correctly predicted as ER-positive and ER-negative tumors, respectively (Supplementary Tables S6, S7).

**Figure 5:**
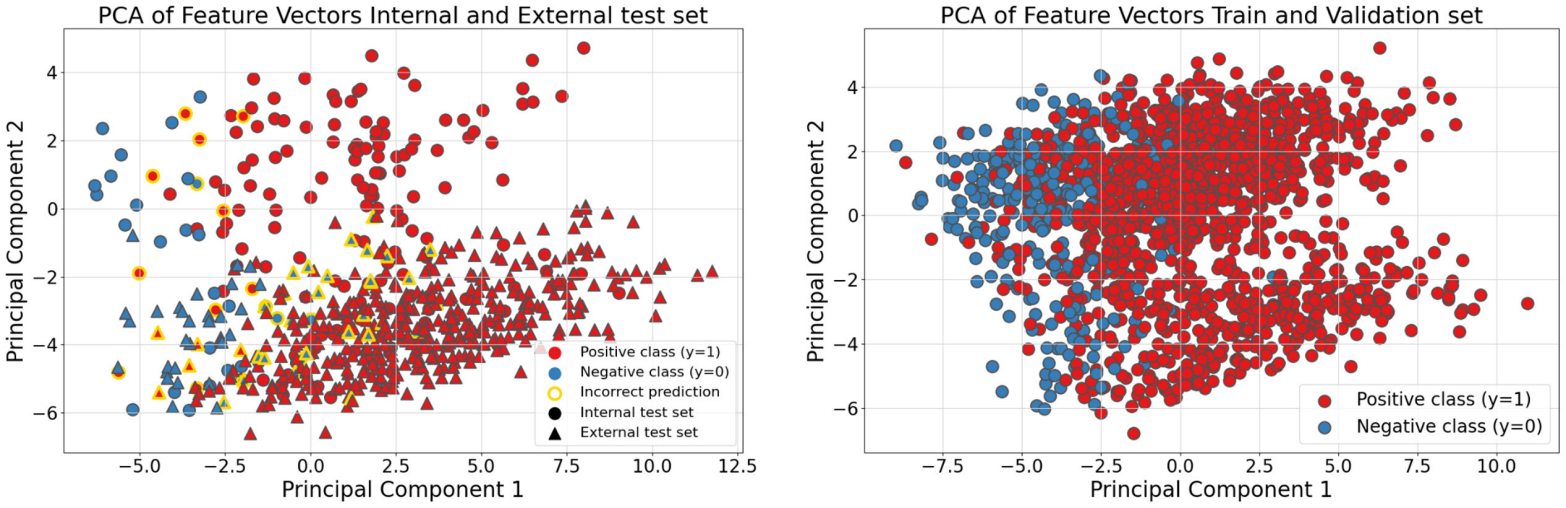
Principal component analysis (PCA). **Left**: result of PCA with two components of the features from the train and validation sets, with the transformation calculated from the training and validation. **Right**: PCA with two components of the features from the two test sets, with the transformation calculated from the training and validation.

The visualization of the learned feature space using PCA did not show a clear separation between the two classes (Figure 5). However, in the training and validation data, two distinct clusters were observed, independent of class labels. In addition, differences between the test sets became apparent, with the internal and external test samples forming separate upper and lower clusters, mainly separated by the second principal component. The visualization also indicated that class separation was less pronounced in the external test set compared to the internal test set (Figure 5).

## 4 Discussion

In this study, CLAM (27) was used to predict ER status in scanned images of HE-stained TMA slides from breast cancer tumors. Patch sizes and CLAM classification heads were compared. The best performing model achieved a macro accuracy, ER-negative accuracy, and ER-positive accuracy of 0.86, 0.79, and 0.93, respectively, on the internal test set. On the external test set, the corresponding results were 0.76, 0.53, and 0.99, respectively.

The proposed model achieved an AUC and micro accuracy of 0.91 and 0.93, respectively, on the external test set. The performance is similar to the results reported by Wang et al. (34) and Gamble et al. (32). Wang et al. achieved AUCs of 0.88 and 0.92, and accuracies of 0.81 and 0.85 with a multi-label model on two different datasets. Gamble et al. achieved an AUC score of 0.86 (0.84–0.87 confidence interval (CI)). The overall AUC and accuracy are not, however, necessarily an optimal measure when comparing results, as they are influenced by the class distribution of the datasets used.

The models' performances were better on the internal test set than on the external test set. This could be due to differences in the datasets. The distribution of ER-positive and ER-negative tumors was similar in the two test sets, but ER-negative tumors in the external test set had a higher proportion of ductal carcinomas and histological grade II tumors compared to ER-negative tumors in the internal test set. Another difference is that the external test set was a screening cohort, while the internal test comprised patients with clinical and screening-detected cancers. It is shown that screening-detected breast cancers have a higher proportion of ER-positive tumors compared to clinical cancers (56, 57). However, the proportions of ER-positive and ER-negative cancers in our internal and external test sets were similar. The PCA of the features from the test sets also showed a difference between the internal and the external test sets. The features in the internal test set were placed similarly to the majority of the features in the train and validation sets, in the principal component 1-principal component 2 space, while the features in the external test set were placed similarly to a minority of the features in the train and validation sets. The features of the external test set also had more overlap of the ER-positive and ER-negative tumors in principal component one. The PCA also showed that with two main components, a clear separation between ER-negative tumors and ER-positive tumors was not found even in the training data, though there was a trend in component one. It is possible, however, that if more components were included in the illustration, a better separation between the classes could be demonstrated with PCA.

Since the model likely uses morphological features in the tissue to predict ER status, differences in histological subtype and histological grade between the test sets could influence the results. To counter this, one could have added random augmentation to the data or stain normalization, prior to the patching and feature extraction, or included data from multiple laboratories in the training and validation sets.

Breast cancer is known for its morphological heterogeneity, and to cover all variations, a larger dataset may be needed to improve the results. Some morphological features associated with ER-negative tumors are typically found near the tumor border, such as pushing tumor margins and infiltration of lymphoid cells, typically seen in medullary carcinomas. TMAs may not cover these areas of the tumor, and including WSIs could be necessary to improve the model performance. On the other hand, pushing margins are often seen in medullary carcinomas, which also have other characteristic morphological features presented in TMAs, such as sheets of tumor cells instead of tubular structures. It is also possible that WSIs could introduce redundant information, and more variance, and one might have to adjust for imbalance in the tissue between different slides. The use of WSIs would also lead to much more data, which would increase training time.

Most breast cancers are ER-positive. ER-positive cancers were also overrepresented in our dataset. Weighting during the hyperparameter optimization and CLAM's weighted sampling scheme were used to counter the class imbalance. However, the model still performed worse on ER-negative cancers than ER-positive cancers. In the clinic, correct assessment of ER status is of great importance for prognostication and treatment of breast cancer patients. In general, ER-positive tumors are associated with a better prognosis than tumors that are ER-negative (9). Patients with ER-positive tumors will most likely be given antihormonal treatment (2), and such treatment is shown to improve prognosis for this subgroup. False-positive ER prediction may lead to unnecessary hormonal treatment, with a low likelihood of effect, but with a risk of unwanted side effects. On the other hand, a false-negative ER prediction may lead to missed hormonal therapy, which is also an undesired scenario for the patient. The proposed model predicted ER-positive tumors with a higher class accuracy than ER-negative tumors. It produced few false-negative predictions (incorrect ER-negative predictions) on both test sets.

In this study, two patch sizes were tested. The models trained with patch size 1,024 × 1,024 achieved better results on ER-negative tumors than those trained with patch size 256 × 256, indicating that a larger context may be necessary when predicting ER status in HE-stained slides. Patch size 2,048 × 2,048 was evaluated, without noticeable improvement. However, one could argue that it may be necessary to investigate even more extreme patch sizes, or keep the same patch size, but extract TMA cores at different magnifications. It is also possible that a multi-scale model, including both a larger context and local information, would improve the results.

The model predicted ER-positive tumors well but struggled more with ER-negative samples. A larger, more balanced dataset could potentially improve the model's performance on the ER-negative tumors. As breast cancer is heterogeneous, and IHC is needed for visualization of estrogen receptors, correct prediction of ER status in HE-stained slides based on the analysis of tissue morphology may need a larger and more diverse dataset. In this study, we wanted to predict ER status from images alone. However, if morphological patterns covered by tabular data such as histological subtype and histological grade are the main contributors to the model's decision, it would be interesting to compare the results of our image-based model with a model trained on tabular data. However, tabular data describing patterns such as tumor-infiltrating lymphocytes, pushing margins, and necrosis are not available in standard pathology reports, and thus favor an image-based model. Furthermore, image-based models could possibly decrease pathologists' workload more than models based on tabular data, since tabular data such as histological subtype and grade would have to be determined by a pathologist.

A main limitation in this study is the class imbalance in the dataset, with a high proportion of ER-positive tumors. Although a high proportion of ER-positive tumors is also found in the clinic, the model may have performed better if we had a higher proportion of ER-negative tumors in our dataset. All slides were stained and scanned at NTNU. The model may have been more robust if we had included slides stained and scanned at other laboratories in the training data. A test set stained and scanned at another laboratory would also have strengthened the study, and we would have been able to assess generalizability even better.

A main strength in this study is the use of internal and external test sets, allowing for a more robust assessment of generalizability. The samples were divided on patient level, and to allow fair comparison between the experiments, the same data split was used for all experiments. More than 2,000 patients from four different cohorts were included in the study. Extensive hyperparameter tuning was conducted to find the best configuration. Class-wise and micro- and macro-averaged metrics were reported to give different insights into performance. Statistical analysis was performed to further assess generalizability and to assess which component of the model affected performance. Principal component analysis was also used to further enhance the understanding of the performance differences between the datasets.

## 5 Conclusion

The proposed method classified breast cancer tumors as either ER-positive or ER-negative. The highest accuracy was achieved for ER-positive tumors. Two patch sizes were compared, and the results were significantly better with the large patch size than the small patch size. While the model's results are good on ER-positive tumors, further studies are needed to generalize to new datasets and improve performance on ER-negative tumors.

## Data Availability

The datasets presented in this article are not readily available because the dataset generated and/or analyzed during the current study is not publicly available according to the ethical approval for this study. Requests to access the datasets should be directed to marit.valla@ntnu.no.
