# Supplementary material for: Predicting estrogen receptor status from HE-stained breast cancer slides using artificial intelligence
Source: Front Med (Lausanne). 2025 Jun 9;12:1593143. doi: 10.3389/fmed.2025.1593143 (PMC12183256; doi:10.3389/fmed.2025.1593143)
Supplement: Supplementary file 1 [file Data_Sheet_1.pdf]

Supplementary Material

1 SUPPLEMENTARY DATA

This document contains an additional table related to the manuscript entitled ”Predicting estrogen receptor status from HE-stained breast cancer slides using artificial intelligence”.

2 SUPPLEMENTARY TABLES AND FIGURES

SUPPLEMENTARY MATERIAL

The weighted accuracy was calculated using 0.8 and 0.2 as weights for the er negative and er positive classes, respectively (Equation S1-S2).

The ER-positive and ER-negative cases stratified by histological subtype and histological grade in the BCS-1, BCS-2, and BCS-3 cohorts and in the two test sets are presented in Table S1-S2.

The six classification heads used in the experiments is presented in Table S3, and the hyperparameter ranges tested for with Optuna in Table S4.

The values for the custom preset file is presented in Table S5.

The results for the resulting model from each experiment on two test sets are presented in Table S6.

The hyperparameters for the multi-branch CLAM model with nano classification head, trained and evaluated on patches of size 1024×1024. and hyperparameters for all experiments for all experiments are presented in Table S8 and S9.

$$ACC_{weighted} = \sum_{i=1}^{N} w_i \cdot ACC_i \tag{S1}$$

$$ACC_{macro} = \frac{1}{N} \sum_{i=1}^{N} ACC_i \tag{S2}$$

Table S1. The number, and percentage, of cases of each histological subtype and grade included in BCS-1, BCS-2, and BCS-3 for er positive and er negative cases.

	ER positive (N) (%)	ER negative (N) (%)	Total (N) (%)
**Histological subtype**			
Ductal	1150 (78.1)	196 (67.8)	1346 (76.4)
Lobular	177 (12.0)	16 (5.5)	193 (11.0)
Medullary	20 (1.4)	39 (13.5)	59 (3.3)
Mucinous	56 (3.8)	2 (0.7)	58 (3.3)
Papillary	32 (2.2)	4 (1.4)	36 (2.0)
Metaplastic	2 (0.1)	14 (4.8)	16 (0.9)
Tubular	5 (0.3)	0	5 (0.3)
Other	30 (2.0)	18 (6.2)	48 (2.7)
Missing	1 (0.1)	0	1 (0.1)
**Histological grade**			
I	232 (16.8)	9 (3.1)	241 (13.7)
II	840 (57.0)	62 (21.5)	902 (51.2)
III	399 (27.1)	218 (75.4)	617 (35.0)
Missing	2 ()	0 ()	2 (0.1)
**Total**	1473 (100.0)	289 (100.0)	1762 (100.0)

Table S2. Histological subtype and grade in the internal and external test sets according to estrogen receptor (ER) status.

	**Internal test set**			**External test set**		
	ER positive (N) (%)	ER negative (N) (%)	Total (N) (%)	ER positive (N) (%)	ER negative (N) (%)	Total (N) (%)
**Histological subtype**						
Ductal	116 (78.9)	20 (69.0)	136 (77.3)	334 (83.7)	49 (83.1)	383 (83.6)
Lobular	10 (6.8)	0 (0.0)	10 (5.7)	46 (11.5)	2 (3.4)	48 (10.5)
Medullary	2 (1.4)	5 (17.2)	7 (0.6)	0 (0.0)	4 (6.8)	4 (0.9)
Mucinous	9 (6.1)	0 (0.0)	9 (5.1)	13 (3.3)	1 (1.7)	14 (3.1)
Papillary	6 (4.1)	0 (0.0)	6 (3.4)	0 (0.0)	0 (0.0)	0 (0.0)
Metaplastic	0 (0.0)	1 (3.4)	1 (0.6)	0 (0.0)	0 (0.0)	0 (0.0)
Tubular	1 (0.7)	0 (0.0)	1 (0.6)	6 (1.5)	0 (0.0)	6 (1.3)
Other	3 (2.0)	3 (10.3)	6 (3.4)	0 (0.0)	3 (5.1)	3 (0.7)
Missing	0 (0.0)	0 (0.0)	0 (0.0)	0 (0.0)	0 (0.0)	0 (0.0)
**Histological grade**						
I	24 (16.3)	1 (3.4)	25 (14.2)	184 (0.46)	2 (3.4)	186 (40.6)
II	81 (55.1)	7 (24.1)	88 (50.0)	177 (44.4)	19 (32.2)	196 (42.8)
III	41 (27.9)	21 (72.4)	62 (35.2)	38 (9.5)	38 (64.4)	76 (16.6)
Missing	1 (0.7)	0 (0.0)	1 (0.6)	0 (0.0)	0 (0.0)	0 (0.0)
**Total**	147 (100.0)	29 (100.0)	176 (100.0)	399 (100.0)	59 (100.0)	458 (100.0)

Table S3. Size of classification heads.

Name	Size
big	[1024, 512, 384]
small	[1024, 512, 256]
mini	[1024, 512, 256, 128]
micro	[1024, 512, 256, 128, 64]
nano	[1024, 512, 128, 64, 32]
pico	[1024, 512, 128, 32, 16]

Table S4. Hyperparameter ranges searched for with Optuna. B represent the number of positive and negative patches sampled for CLAM for the instance clustering. Abbreviations: ADAM: Adaptive Moment Estimation, SGD: Stochastic gradient descent, SVM: Support vector machine, CE: Cross-entropy.

Hyperparameter	Range
Learning rate	$1x10^{-5}$ - $1x10^{-3}$ (log scale)
Regularization, L2	$1x10^{-6}$ - $1x10^{-4}$ (log scale)
Optimization	ADAM, SGD
Dropout rate	0-0.5, 0.05 (increments 0.05)
Bag loss	SVM, CE
Instance level clustering	True, False
Instance level clustering loss function	SVM, CE, None
Bag weight	0.5 - 1.0 (increments 0.05)
Clustering bag size, B	4, 8, 16, 32, 64, 128

Table S5. Values used in custom preset file for tissue segmentation and patch generation in CLAM.

Parameter	Value
seg_leve	-1
sthresh	2
mthresh	7
close	4
use_otsu	FALSE
a_t	8
a_h	8
max_n_holes	64
vis_level	-1
line_thickness	100
white_thresh	5
black_thresh	50
use_padding	TRUE
contour_fn	four_pt
keep_ids	none
exclude_ids	none

Table S6. AUC, accuracy, and ER negative (class 0) and ER positive (class 1) accuracy for the resulting model from each experiment on the internal and external test set. Abbreviations: ACC = accuracy, mACC = macro accuracy, wACC = weighted accuracy, AUC = Area under the curve.

Method	Patch size	Classification head	Dataset	ACC 0	ACC 1	ACC	wACC	mACC	AUC
sb	256	big	internal	0.55	0.95	0.88	0.63	0.75	0.93
sb	256	small	internal	0.52	0.97	0.90	0.61	0.75	0.92
sb	256	mini	internal	0.62	0.95	0.89	0.69	0.78	0.93
sb	256	micro	internal	0.59	0.96	0.90	0.66	0.77	0.93
sb	256	nano	internal	0.62	0.93	0.88	0.68	0.77	0.91
sb	256	pico	internal	0.69	0.92	0.88	0.74	0.80	0.92
mb	256	big	internal	0.66	0.93	0.88	0.71	0.79	0.91
mb	256	small	internal	0.62	0.95	0.89	0.69	0.78	0.92
mb	256	mini	internal	0.62	0.92	0.87	0.68	0.77	0.92
mb	256	micro	internal	0.62	0.93	0.88	0.68	0.77	0.92
mb	256	nano	internal	0.62	0.93	0.88	0.68	0.77	0.92
mb	256	pico	internal	0.59	0.94	0.88	0.66	0.76	0.92
sb	1024	big	internal	0.72	0.95	0.91	0.77	0.83	0.95
sb	1024	small	internal	0.76	0.95	0.91	0.80	0.85	0.95
sb	1024	mini	internal	0.76	0.94	0.91	0.79	0.85	0.95
sb	1024	micro	internal	0.76	0.95	0.91	0.80	0.85	0.95
sb	1024	nano	internal	0.76	0.94	0.91	0.79	0.85	0.95
sb	1024	pico	internal	0.76	0.95	0.91	0.80	0.85	0.95
mb	1024	big	internal	0.69	0.94	0.90	0.74	0.81	0.95
mb	1024	small	internal	0.72	0.95	0.91	0.77	0.83	0.95
mb	1024	mini	internal	0.72	0.95	0.91	0.77	0.83	0.95
mb	1024	micro	internal	0.76	0.95	0.91	0.80	0.85	0.95
mb	1024	nano	internal	0.79	0.93	0.91	0.82	0.86	0.95
mb	1024	pico	internal	0.62	0.95	0.90	0.69	0.79	0.94
sb	256	big	external	0.31	0.99	0.90	0.40	0.65	0.90
sb	256	small	external	0.32	0.99	0.91	0.39	0.66	0.89
sb	256	mini	external	0.34	0.99	0.91	0.39	0.66	0.90
sb	256	micro	external	0.34	0.99	0.91	0.40	0.67	0.90
sb	256	nano	external	0.32	0.99	0.90	0.38	0.65	0.89
sb	256	pico	external	0.34	0.99	0.91	0.39	0.66	0.89
mb	256	big	external	0.32	0.99	0.91	0.39	0.66	0.89
mb	256	small	external	0.36	0.99	0.91	0.41	0.68	0.90
mb	256	mini	external	0.34	0.99	0.91	0.40	0.67	0.89
mb	256	micro	external	0.32	0.99	0.91	0.39	0.66	0.89
mb	256	nano	external	0.34	0.99	0.91	0.40	0.67	0.89
mb	256	pico	external	0.36	0.99	0.91	0.41	0.67	0.89
sb	1024	big	external	0.49	0.99	0.93	0.49	0.74	0.92
sb	1024	small	external	0.54	0.98	0.93	0.52	0.76	0.92
sb	1024	mini	external	0.49	0.99	0.92	0.49	0.74	0.91
sb	1024	micro	external	0.54	0.98	0.93	0.52	0.76	0.92
sb	1024	nano	external	0.51	0.99	0.93	0.51	0.75	0.92
sb	1024	pico	external	0.49	0.99	0.92	0.49	0.74	0.91
mb	1024	big	external	0.47	0.99	0.92	0.49	0.73	0.91
mb	1024	small	external	0.51	0.98	0.92	0.51	0.75	0.91
mb	1024	mini	external	0.49	0.99	0.92	0.49	0.74	0.92
mb	1024	micro	external	0.56	0.99	0.93	0.52	0.77	0.92
mb	1024	nano	external	0.53	0.99	0.93	0.52	0.76	0.91
mb	1024	pico	external	0.34	0.99	0.91	0.39	0.66	0.90

Table S7. True positive, false positive, true negative, and false negative cases predicted with the mb clam model with the nano classification head (patch size 1024×1024) on the internal and external test sets. Abbreviations: TP: true positives, FP: false positives, TN: true negatives, FN: false negatives.

	Internal test set				External test set			
	TP	FP	TN	FN	TP	FP	TN	FN
**Histological subtype**								
Ductal	108	5	15	8	331	25	24	3
Lobular	10	0	0	0	46	1	1	0
Medullary	1	0	5	1	0	1	3	0
Mucinous	8	0	0	1	11	1	0	2
Papillary	6	0	0	0	0	0	0	0
Metaplastic	0	0	1	0	0	0	0	0
Tubular	1	0	0	0	6	0	0	0
Other	3	1	2	0	0	0	3	0
Missing	0	0	0	0	0	0	0	0
**Histological grade**								
I	23	0	1	1	183	2	0	1
II	80	3	4	1	176	9	10	1
III	33	3	18	8	35	17	21	3
Missing	1	0	0	0	0	0	0	0
**Total**	137	6	23	10	394	28	31	5

Table S8. Hyperparameter for the multi-branch CLAM with nano classification head, trained, and evaluated on patches of size 1024×1024. Abbreviations:

Hyperparameter	Value
Learning rate	1.0170e-05
Regularization, L2	5.5637e-05
Optimization	SGD
Drop out	0.05
Bag loss	SVM
Instance level clustering	True
Instance level clustering loss function	CE
Bag weight	0.8
Clustering Bag size, B	8

Table S9. Hyperparameter selection for experiments. Abbreviations: lr: Learning rate, reg: regularization, L2, opt: optimization, inst loss: instance level clustering loss function, B: clustering Bag size.

Experiment	lr	reg	Bag loss	Dropout	opt	Bag weight	inst loss	B
256 sb big	1.4e-05	6.1e-05	SVM	0.40	SGD	0.75	SVM	8
256 sb small	3.3e-05	2.6e-05	CE	0.05	SGD	0.55	—	16
256 sb mini	1.2e-05	1.0e-06	SVM	0.30	SGD	0.50	SVM	8
256 sb micro	1.4e-05	3.1e-05	SVM	0.05	SGD	0.70	—	16
256 sb nano	1.2e-05	1.4e-05	CE	0.45	SGD	1.00	—	32
256 sb pico	1.0e-05	9.0e-05	SVM	0.45	SGD	0.95	SVM	32
256 mb big	1.0e-05	3.2e-06	SVM	0.40	SGD	0.70	—	256
256 mb small	1.5e-05	3.9e-05	SVM	0.20	SGD	0.70	—	128
256 mb mini	1.4e-05	8.0e-06	SVM	0.30	SGD	0.65	SVM	32
256 mb micro	1.1e-05	6.4e-06	SVM	0.35	SGD	0.95	—	128
256 mb nano	1.2e-05	5.4e-06	SVM	0.05	SGD	0.90	—	64
256 mb pico	1.4e-05	1.2e-05	SVM	0.30	SGD	0.55	SVM	16
1024 sb big	1.7e-05	1.3e-05	SVM	0.10	SGD	0.90	CE	32
1024 sb small	1.1e-05	9.8e-05	SVM	0.10	SGD	0.50	CE	4
1024 sb mini	1.1e-05	5.6e-06	CE	0.15	SGD	0.85	SVM	8
1024 sb micro	1.1e-05	1.5e-06	SVM	0.00	SGD	0.65	CE	8
1024 sb nano	1.0e-05	2.3e-06	SVM	0.20	SGD	0.65	SVM	32
1024 sb pico	2.0e-05	1.1e-05	CE	0.50	SGD	0.60	—	64
1024 mb big	1.3e-05	3.5e-06	SVM	0.20	SGD	0.80	—	64
1024 mb small	1.7e-05	5.4e-05	SVM	0.45	SGD	0.95	—	32
1024 mb mini	1.1e-05	1.5e-05	SVM	0.10	SGD	0.75	—	256
1024 mb micro	1.2e-05	1.5e-05	CE	0.30	SGD	0.85	SVM	128
1024 mb nano	1.0e-05	5.6e-05	SVM	0.05	SGD	0.80	CE	8
1024 mb pico	4.2e-05	3.0e-06	CE	0.35	SGD	0.65	CE	4
